# Increased intestinal permeability and tight junction disruption by altered expression and localization of occludin in a murine graft versus host disease model

**DOI:** 10.1186/1471-230X-11-109

**Published:** 2011-10-06

**Authors:** Rainer Noth, Julia Lange-Grumfeld, Eckhard Stüber, Marie-Luise Kruse, Mark Ellrichmann, Robert Häsler, Jochen Hampe, Burkhard Bewig, Philip Rosenstiel, Stefan Schreiber, Alexander Arlt

**Affiliations:** 1 Department of Internal Medicine, University Hospital Schleswig-Holstein, Campus Kiel, 24105 Kiel, Germany; 2 Institute of Clinical Molecular Biology, University Hospital Schleswig-Holstein, Campus Kiel, 24105 Kiel, Germany

## Abstract

**Background:**

Hematopoietic stem cell transplantation is increasingly performed for hematologic diseases. As a major side effect, acute graft versus host disease (GvHD) with serious gastrointestinal symptoms including diarrhea, gastrointestinal bleeding and high mortality can be observed. Because surveillance and biopsies of human gastrointestinal GvHD are difficult to perform, rare information of the alterations of the gastrointestinal barrier exists resulting in a need for systematic animal models.

**Methods:**

To investigate the effects of GvHD on the intestinal barrier of the small intestine we utilized an established acute semi allogenic GvHD in C57BL/6 and B6D2F1 mice.

**Results:**

By assessing the differential uptake of lactulose and mannitol in the jejunum, we observed an increased paracellular permeability as a likely mechanism for disturbed intestinal barrier function. Electron microscopy, immunohistochemistry and PCR analysis indicated profound changes of the tight-junction complex, characterized by downregulation of the tight junction protein occludin without any changes in ZO-1. Furthermore TNF-α expression was significantly upregulated.

**Conclusions:**

This analysis in a murine model of GvHD of the small intestine demonstrates serious impairment of intestinal barrier function in the jejunum, with an increased permeability and morphological changes through downregulation and localization shift of the tight junction protein occludin.

## Background

Hematopoietic stem cell transplantation is an established treatment modality for a wide range of hematologic malignancies [1]. After bone marrow transplantation, T cells present in the graft, either as contaminants or intentionally introduced into the host, can attack the host tissues of the transplant recipient by recognition of host tissues as foreign antigens. The T cells produce an excess of cytokines like TNF-α and interferon-gamma (IFNγ) which in turn lead to a pro-inflammatory environment. This graft-versus-host-disease (GvHD) affects up to 70% of patients who undergo stem cell transplantation and is clinically divided into acute and chronic forms which also involve different immune cell subsets and cytokine profiles [1-4].

The *acute *form of the disease is mostly observed within the first 100 days post-transplantion, and is a major challenge to transplants owing to associated morbidity and mortality [1,5]. Classically, acute GvHD is characterized by selective damage to liver, skin and the gastrointestinal (GI) tract. The manifestation in the GI tract (GI GvHD) can result in severe diarrhea, abdominal pain, nausea, and vomiting. The gastrointestinal manifestation of the disease is typically diagnosed via intestinal biopsy. However, risk of endoscopy is higher in these ill patients and frequently thrombocytopenia complicates mucosal biopsies [6]. Histological findings that support the diagnosis are nonspecific and have limited sensitivity and specificity [2]. In addition, specific options for treatment of GvHD of the intestine [3,6] are currently lacking. The problem in the diagnosis and treatment of GI GvHD are due to the still limited understanding of the pathophysiology of the disease. However it is well established that disruption of the mucosal barrier leads to the GI-symptoms and the increase in morbidity and mortality [2,3].

Murine acute GVHD has long been investigated for two reasons: as an animal model for the immune pathomechanisms of human GVHD after allogenic bone marrow transplantation. Furthermore, due to its characteristic changes in the intestinal histology consisting of lymphocytic infiltrates, crypt hyperplasia, and villous atrophy, it serves as a model for immunologically mediated atrophic-hyperregenerative diseases of the intestine (29, 30). By improving the understanding of the pathophysiology of GI GVHD more focused and effective diagnostic and therapeutic options could be implemented leading to reduced morbidity and mortality.

The aim of this study was to evaluate effects of a GI GvHD in a mouse model regarding the functions of the gastrointestinal barrier by analysing the intestinal permeability and the composition of the tight junctions. Our analysis showed a seriously impaired intestinal barrier function with an increased permeability associated with an upregulation of TNF-α expression and downregulation and localization shift of the tight junction protein occludin.

## Methods

### Animal model

C57BL/6, DBA/2 mice and the F1 intercross of these two mouse strains (B6D2F1) were raised and kept under standard conditions in the animal facility of the University Hospital of Kiel, Germany. Animal care and experiments were approved by the local commitee for animal use (Az 28-5/96).

To induce semi-allogenic graft-versus host disease, a slightly modified procedure as described by [7] was performed. To prepare donor lymphocytes, spleen and mesenterial lymph nodes of C57BL/6 mice were removed and pressed through a cell filter (40 μm pore size). Red blood cells were subsequently lysed by a hypotonic lysing buffer (ACK-buffer, Boehringer Ingelheim, Germany) The resulting lymphocytes (80 × 10^6 ^cells/animal) were transferred to 8-14-week-old irradiated (7,5 Gy) B6D2F1 mice of the same sex by intraperitoneal injection. Six days after the induction of semi-allogenic GvHD, recipient animals were killed. The small bowel (jejunum) was removed and frozen in liquid nitrogen or fixed in 10% phosphate buffered (pH 7.4) formalin for further analysis. Irradiated B6D2F1 mice, which were transferred with the same amount of syngenic (B6D2F1) cells served as controls.

### Measurement of intestinal permeability

Intestinal permeability was determined using two non-metabolized sugars. Threehundred mililiters lactulose and 200 mg mannitol were dissolved in 20 ml distilled water. After a fasting period of 12 hours, GvHD-mice and control animals received 1 ml of the lactulose/mannitol solution by orogastral tube. One hour after feeding, blood was taken from the sinus cavernosus under anaesthesia with ether. Serum concentrations of lactulose and mannitol were determined using melibiose as internal standard as described below.

### Measurement of lactulose and mannitol serum concentrations

Mannitol and the disaccharide lactulose, (4-0-ß-D-galactopyranosyl-D-fructofuranose) for oral administration were obtained from Fluka (Seelze, Germany) and Calbiochem (San Diego, Ca, USA) while melibiose for internal standards was supplied from Sigma (Deisenhofen, Germany). The high-performance-liquid-chromatography (HPLC) equipment was from Merck (Darmstadt, Germany) and comprised an eluent degas module, a micro injection valve fitted with a 25 μl loop and a gradient pump. The pulsed amperometric detector (PAD) with a gold electrode and a silver/silver chloride reference electrode, a Carbopac^® ^PA 100 anion exchange column and a guard column filled with the same material was from Dionex (Idstein, Germany). Data were processed using a Merck integrator D7500. Deionised water with high resistance (18 M Ώ/per XYZ) for dilution of samples and eluent was from Merck.

### Eluent preparation

High-performance-liquid-chromatography water was degassed by sparkling with helium for 15 minutes. Fifty percent sodium hydroxide solution was added and gently stirred for 5 minutes to achieve the required concentration of 90 mM NaOH.

### Sample preparation (serum)

Serum was separated from whole-blood samples by a 10 minute centrifugation at 3000 rpm and was stored afterwards at -20°C for up to two months until analysis. An aliquot of 0.5 ml serum was deproteinised by precipitation with 5% 5-sulfosalicylic acid and 200 μl of the internal standard (melibiose 0.1 mg/l) was added. After a 15 minutes incubation period (4°C), the samples were centrifuged at 9000 rpm for 5 minutes and the supernatants were removed and diluted (1:100) with 90 mmol/l NaOH. Twenty five microliters of this supernatant were then applied onto the anion exchange column.

### Analyses of carbohydrates with HPLC and pulsed amperometric detection (PAD)

The samples were eluted with the 90 mmol NaOH eluent at a flow rate of 1 ml/min at 25°C. Analytes were separated through the Carbopac^® ^100 anion exchange column and detected with a pulsed amperometric detector (PAD) using a gold working electrode and a silver/silver chloride reference electrode. A repeating sequence of three different potentials was used. Carbohydrates were detected by measuring the electrical current generated by their oxidation at the surface of a gold electrode (E1: 0.05 V, T1: 0.4 sec.). The second potential oxidized the gold electrode to clean the surface (E2: 0.75 V, T2 0.2 sec.). The third potential reduced the gold oxide on the electrode surface back to gold, thus permitting a new detection during the next cycle (E3: -0.15 V, T3: 0.4 sec). Sensitivity on the PAD was set to 100 nC. Calculation of sugar concentration was performed by integrator analysis of the area of the melibiose reference peak and the area of the mannitol and lactulose peaks.

### Electron microscopy

The jejunum specimens were fixed for at least four hours in Karnovsky solution (2.5% glutaraldehyde and 2% paraformaldehyde in 0.1 M sodium-cacodylate-buffer, pH 7.0). The fixed samples were washed three times for ten minutes with a 0.1 M sodium-cacodylate-buffer (pH 7.0) followed by a second fixation in 1% cacodylate-buffered osmiumtetroxide solution (Roth, Karlsruhe, Germany). Dehydration with ethanol was followed by imbedding with Araldit (Serva, Germany). For contrasting, uranylacetate treatment of the ultrathin sections was performed subsequently. A Zeiss electron microscope (EM 902, Zeiss, Jena, Germany) was used for imaging.

### Immune fluorescence microscopy

Snap-frozen jejunum samples were cut into 1 μm sections at -24°C. These sections were dried, fixed with acetone and rehydrated in phosphate-buffered saline (Dulbecco's PBS (1×), PAA Laboratories GmbH, Austria). To suppress unspecific background, fluorescence blocking reagent Vectastain EliteABC-Kit (Rabbit IgG, Vector Laboratories, Servion, Switzerland) was applied followed by incubation with the primary antibody rabbit-anti-occludin and rabbit-anti-ZO-1 (Zymed Laboratories, USA). After washing with PBS, incubation with the fluorescent secondary antibody Alexa Fluor^® ^488 anti-rabbit (Invitrogen™, Molecular Probes™, Oregon, USA) followed. After washing with PBS, Phalloidin (Sigma) was applied as a cytoskeleton dye. For staining of nucleic acids, DAPI was used (Sigma, USA). Imaging was performed with the fluorescence microscope Axion imager Z.1 (Carl Zeiss MicroImaging GmbH, Jena, Germany) and analysed using theAxion Vision (Zeiss) software.

### Expression analysis

#### RNA isolation and cDNA synthesis

Total RNA was extracted from frozen biopsies using silica gel-based spin columns (RNeasy Kit; QIAGEN, Hilden, Germany). Genomic DNA was digested by treatment with deoxyribonuclease I (QIAGEN). Reverse transcription of 2 mg RNA was performed using 0.5 μg oligo (dT)15-primers (You prime First strand cDNA synthesis kit; Amersham Biosciences, Freiburg, Germany) according to the manufacturer's instructions.

#### Primers

Specific intron-spanning primers for TNF-α, occludin and ZO-1 were designed using the Primer3 software [8] Oligonucleotides were obtained from Sigma ARK (Darmstadt, Germany). The oligonucleotide sequences are depicted as followed: 5'-TGA AGG TCG GTG TGA ACG GAT -3 (G3PDH fwd, GenBank Accession # NM_002046) and 5'-CAT GTA GGC CAT GAG GTC CAC -3' (G3PDH rev'); 5'-AGA CTA CAC GAC AGG TGG GG -3' (Occludin fwd, NM_008756) and 5'-CTG CAG ACC TGC ATC AAA AT -3' (Occlucin rev); 5'-GCA GAC TTC TGG AGG TTT CG -3' (ZO-1 fwd, NM_009386) and 5'-CTT GCC AAC TTT TCT CTG GC -3' (ZO-1 rev); 5'-TCT ACT GAA CTT CGG GGT GA -3'(TNF-α fwd, NM_013693) and 5'-CAC TTG GTG GTT TGC TAC GA -3' (TNF-α rev)

#### Polymerase chain reaction (PCR)

PCR was performed with Gene Amp^® ^PCR system 9700 (Applied Biosystems, USA) using two microliters of cDNA dissolved in H_2_O, primer F/R (1.0 μl), GoTaq DNA-Polymerase (0.1 μl), dNTP mix (0.2 μl), 5 × Green GoTaq Reaction Buffer (4.0 μl) and autoclaved H_2_O (12.7 μl). Samples were loaded into capillary tubes and incubated in the PCR-machine with initial denaturation at 94°C for 5 min, followed by 32-40 cycles (G3PDH 32, occludin/ZO-1 35, TNF-α 40), each cycle consisting of 94°C for 30 sec. for denaturation, primer specific annealing temperature for 30 sec., and 72°C for 30 sec to one minute for elongation.

### Data analysis

Differences between experimental groups were determined by quantification of the fold change, which was based on the ratio of the medians of the two compared groups. Significance was determined by using the Mann-Whitney U-test, p-values were corrected for multiple testing (here: 8 parameters) using the Benjamini-Hochberg correction.

## Results

### Increased paracellular intestinal permeability

**Figure 1:**
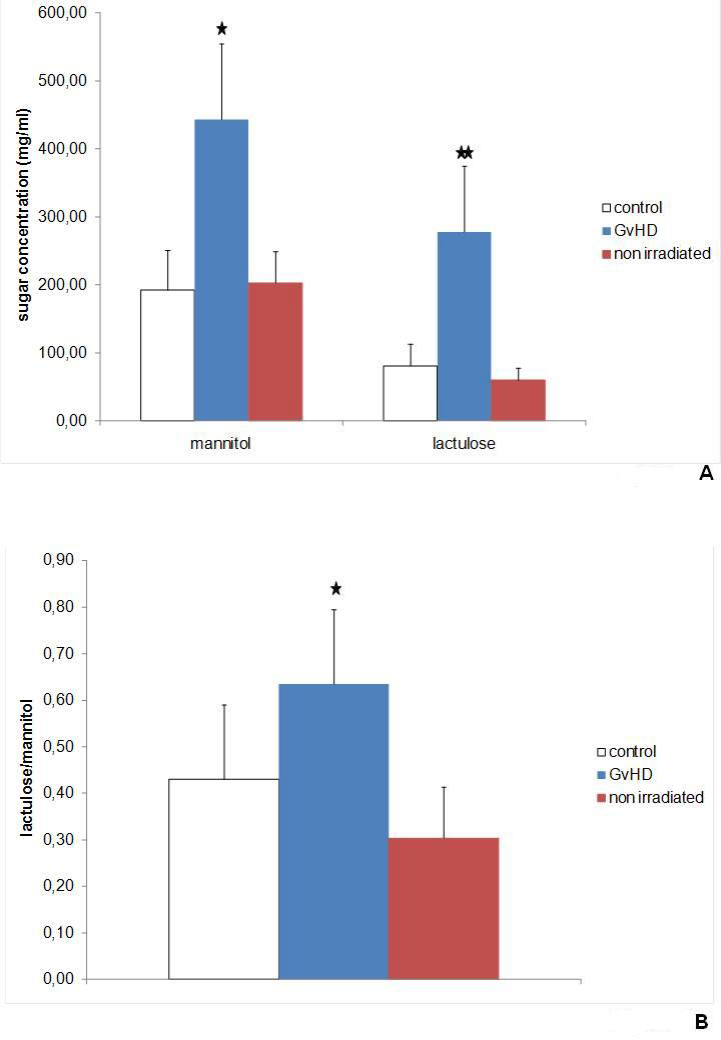
**Gastrointestinal permeability**. Intestinal permeability was determined using lactulose and mannitol. After a fasting period of 12 hours GvHD-mice (n = 20), control animals (n = 18) and non irradiated B6D2F1 mice (n = 8) received 1 ml of the lactulose/mannitol solution by orogastral tube. One hour after feeding serum concentrations of lactulose (representative for paracellular permeability) and mannitol (representative for transcellular permeability) were determined using melibiose as internal standard as described. A) mean and SD of mannitol and lactulose serum concentrations normalized to melbiose. * indicates p-values < 0.05 and ** p-values < 0.01. B) Lactulose/mannitol quotient. * indicates p-value < 0.05.

Six days after induction of the semi allogenic GvHD, intestinal permeability was assessed by differential uptake of lactulose and mannitol in GvHD animals (n = 20) and controls (n = 18). For demonstration of the effects of the irradiation alone differential uptake of lactulose and mannitol of B6D2F1 mice was measured (Figure 1). At this time point the GvHD animals showed a slightly lower weight and are swelling of the intestine in cross anatomy (data not shown).

The measurement of mannitol and lactulose by HPLC analysis showed higher levels of both sugars in comparison to the internal standard melibiose indicating impaired gastrointestinal permeability (Figure 1A). Mannitol levels, an indicator for transcellular permeability, were 443.33 mg/ml in the GvHD animals and 192.44 mg/ml in control animals. The increase in lactulose levels to 277.35 mg/ml in GvHD animals in comparison to 81.18 mg/ml in control animals indicated a higher paracellular permeability in the GvHD group (Figure 1A). By comparing the lactulose to mannitol levels (lactulose/mannitol 0.64 in GvHD vs. 0.43 in control animals, p < 0.05), we were able to show that the disturbance of the paracellular permeability is statistically more pronounced (Figure 1B). Statistical testing by a Man-Whitney U test showed that all observed effects on the gastrointestinal permeability were statistical highly significant (Figure 1A and 1B, p < 0.05).

### GvHD is characterized by alterations of the morphology of the tight junctions

**Figure 2:**
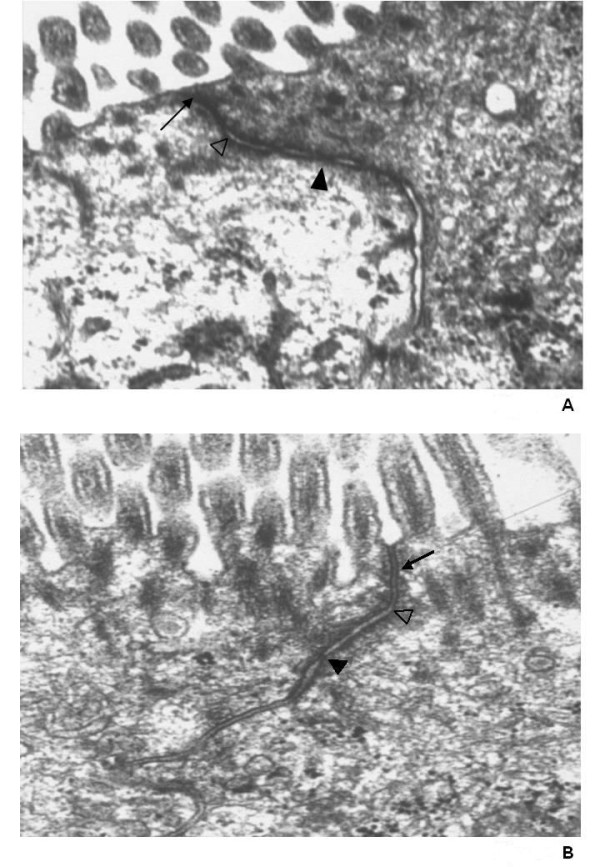
**Electron microscopy of tight junctions of the small intestine**. Morphological analysis of tight junctions of the small intestine of control animals A) and GvHD animals 2B) by electron microscopy. Small black arrow = Tight junction, white triangle = adherens junction, black triangle = desmosome. Representative results of independent examinations of 10 animals each group.

Since permeability testing indicated that GvHD of the small intestine is characterized by an impaired control of the paracellular permeability we next performed morphological analysis of the tight junctions by electron microscopy (Figure 2). In comparison to the control animals (Figure 2A), GvHD led to discontinuous tight-junction strand with widening of the paracellular spaces (Figure 2B).

### GvHD is associated with upregulation of the proinflammatory cytokine TNF-α

**Figure 3:**
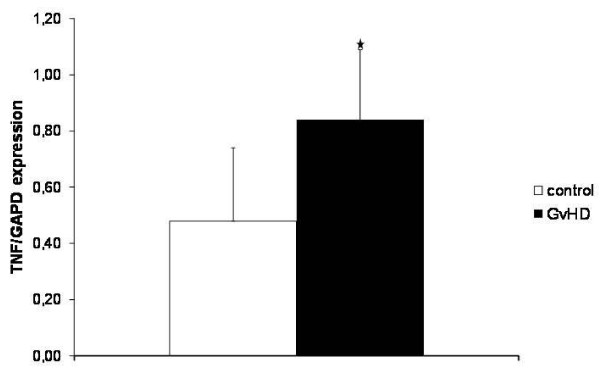
**TNF-α expression in the jejunum**. Expression of the proinflammatory cytokine TNF-α in the specimen of the jejunum of GvHD-mice (n = 20) and control animals (n = 18) was analysed by PCR. Mean and SD of TNF-α expression normalized to the housekeeping gene GAPDH are shown. * indicates p-values < 0.05.

Since TNF-α have been shown to be a critical component of the course of GvHD in general [9] and for the increased paracellular permeability in DSS induced colitis [10], we next analysed expression of this proinflammatory cytokine in specimen of the jejunum by PCR. As shown in Figure 3, GvHD is associated with a strong increase of TNF-α expression at mRNA level in comparison to the expression observed in the jejunum of the control animals.

### Downregulation of occludin but not ZO-1 expression in GvHD

**Figure 4:**
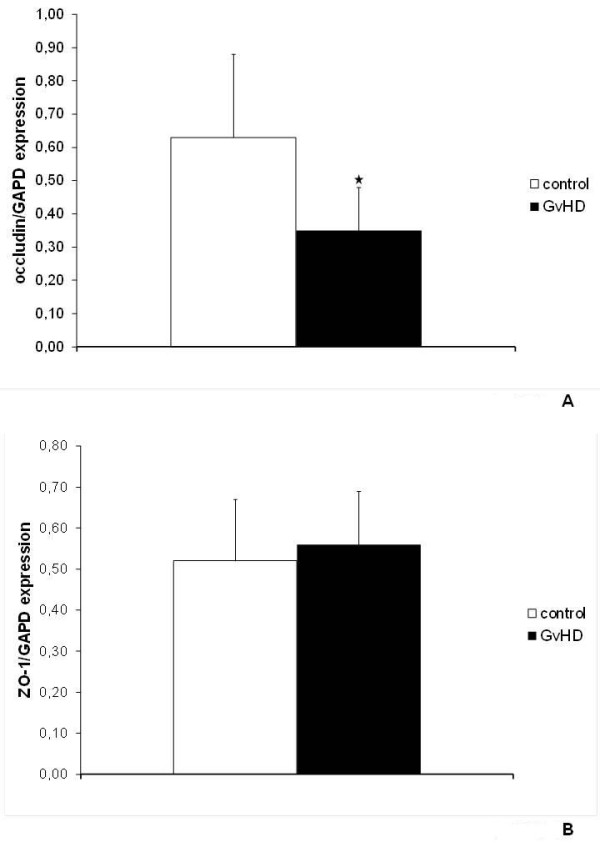
**Occludin and ZO-1 expression in the jejunum**. Expression of the tight junction proteins occludin (A)) and ZO-1 (B)) in the specimen of the jejunum of GvHD-mice (n = 20) and control animals (n = 18) were analysed by PCR. Mean and SD of occludin (A)) and ZO-1 (B)) expression normalized to the housekeeping gene GAPDH are shown. * indicates p-values < 0.05.

The molecular composition of the tight junction by transmembrane and cytoplasmatic proteins is highly complex. Due to their reported involvement in disruption of intestinal barrier function in other disease models, we focused our analysis on the transmembrane protein occludin [11] and the intracellular protein ZO-1 [12]. We showed by rtPCR, that expression levels of occludin were significantly reduced in the course of GvHD (Figure 4A). In contrast, no changes in ZO-1 expression were observed (Figure 4B).

### Changes in intracellular localization of occludin in GvHD

**Figure 5:**
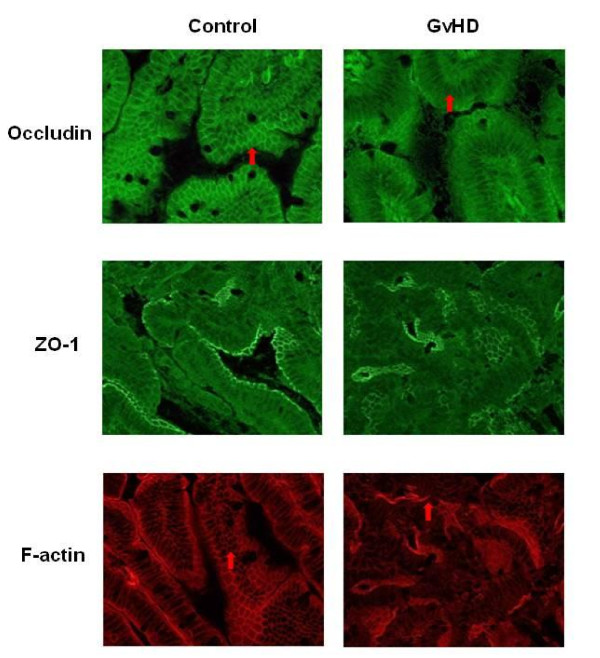
**Intracellular localization of occludin, ZO-1 and F-actin in the jejunum**. Localization of the tight junction proteins occludin (top), ZO-1 (middle) and the actin skeleton protein F-actin (bottom) in jejunum of GvHD-mice (n = 26) and control animals (n = 16) were analysed by immunfluorescence microscopy. Representative results of independent examinations of 10 animals each group. Red arrows highlight alterations in the occludin and F-actin localization.

Besides alterations of expression levels of tight-junction proteins, changes of their intracellular localisation can also affect barrier function, as shown recently [11]. Therefore, indirect immunofluorescence staining for occludin and ZO-1 was performed. Figure 5 shows fluorescence microscopy of control animals (left panel) versus GvHD animals (right panel). Occludin staining was found along the lateral cell walls with enhanced signals at the apical site under control conditions (Figure 5 top left). Tangential sectioning of epithelial cells showed a polygonal pattern reminiscent of mesh wire. In contrast, analysis of GvHD sections revealed a fuzzy appearance of immune reactivity throughout the cell, leaving only the nucleus free of staining indicative of re-localisation from the plasma membrane (Figure 5, top right). Analysis of ZO-1 distribution showed concentrated staining at the apical aspect of the plasma membrane in control slices (Figure 5, middle left) This pattern appeared only slightly impaired in GvHD animals (Figure 5, middle, right) Since a recent report indicated TNF-α induced effects on the interaction of occluding and ZO-1 with components of the actin cytoskeleton [13], the expression of F-actin was analyzed next. Actin is a major constituent of the terminal web at the apical plasma membrane. Underlying the brush border of the epithelium, actin staining apears as a tight line at the apical aspect of the cells, showing the typical mesh wire-like pattern in tangential sections of the epithelial cell layer in control animals (Figure 5, bottom left). In GvDH animals (Figure 5, bottom right) actin staining in epithelial cells appeared disrupted and extended, no longer confined to the terminal web, but mostly diffuse, or randomly accumulated into denser patches of staining.

## Discussion

Acute graft versus host disease (GvHD) is a severe complication after hematopoietic stem cell transplantation. The impaired integrity of the gastrointestinal tract during GvHD (GI-GvHD) seems to play an important role in the amplification of the systemic disease.

In this study, we show for the first time that GI-GvHD leads to tight junction impairment with subsequently increased paracellular permeability. In the murine T-cell mediated acute GI-GvHD model used in this study, the transmembrane tight junction protein occludin is downregulated and its mesh wire-like appearance within the apical tight-junction complex is lost due to cytoplasmic re-localization. Furthermore, we observed an increased expression of TNF-α which might in turn lead to the observed changes in occludin expression and/or localization.

Our findings are in line with other studies on T-cell mediated small bowel diseases which found increased paracellular permeability [14-19]. Tight-junction proteins are essential for the integrity of the intestinal barrier by building the most apical structure and regulating paracellular permeability and polarity of the cell. The tight-junction composition is a highly dynamic process and different tight-junction components can be divided into the transmembrane proteins (occludin, the claudin protein family, junctional adhesion molecules, coxsackie adenovirus receptor and tight-junction associated marvel proteins) and cytoplasmatic proteins (ZO-1 and cingulin). Furthermore, F-actin and myosin, as part of the cytoskeleton [20,21], are involved in the composition of the tight junctions. Interactions between tight-junction proteins, kinase activation and cytokine release influence the regulation of two transepithelial molecular pathways: a high-capacity, charge selective pore pathway for small uncharged molecules influenced primarily by claudin expression and a low-capacity leak pathway for larger ions regardless of charge regulated by the cytoskeleton involving ZO-1 and occludin. We observed a downregulation of occludin and a shift from the membrane to the cytoplasm without any significant changes in ZO-1 expression. Hypothetically, this could be an important cause of the observed increased permeability. Occludin is known to interact with other tight-junction proteins like ZO-1 and F-actin, mediating signal transduction, tissue growth and differentiation as well as cytoskeleton mediated leak pathway regulation [22]. Furthermore, the function and the localisation seem to differ depending on posttranslational phosphorylation by enzymes [20]. Fluorescence recovery after photobleaching analyses showed that occludin is very mobile at the tight-junction undergoing constant remodelling [23]. In line with our observations, recent data in colitis associated impaired barrier function indicated that alterations in the expression level and localization of occludin are pivotal for increased paracellular permeability. Interestingly, the promoter of the occludin gene harbours a TNF-α response element, negatively regulating the transcription of occludin [24]. Referring to the pivotal role of TNF-α in the regulation of gastrointestinal barrier function and permeability [10,13,16,25-28] and the fact that we observed a strong upregulation of TNF-α expression, it is tempting to speculate, that occludin is a central downstream target of TNF-α in GI-GvHD. It is well known, that IFN-γ production by the donor T cells, endotoxins and LPS as a component of the normal gut flora who penetrate the impaired intestinal barrier can lead to an excessive TNF-α production by monocytes and macrophages [4,29-31]. This increased TNF-α expression is associated with the severity of intestinal GvHD [32] and an elegant recent work showed the pivotal role of TNF tight junction remodeling and barrier maintenance [13]. Thus, increased TNF-α may impair the barrier function through disturbance of the tight junction which in turn results in increased paracellular permeability, enforced translocation of antigens from the gut into the circulation and an uncontrolled systemic inflammatory response.

In contrast to colitis model systems in mice [33-35], we did not observe alterations in the cytoplasmatic tight junction protein ZO-1. The alterations in the tight junction complex and its components seem to be dependent of the disease as indicated by data showing alterations in only the claudin family in other model systems [36,37].

By immune fluorescence microscopy, we were able to show that f-actin was released from the tight organisation of the terminal web beneath the apical plasma membrane and showed a mostly diffuse, spreaded appearance with some more densely packed areas in irregular patterns in GvDH. This could be due to a contraction of the cytoskeleton with tension on the tight-junction followed by an augmented paracellular channel as described by other studies [17] or part of the response of intestinal epithelia to TNF-α induced shedding [13] as reported recently.

## Conclusions

In conclusion, the present study demonstrates that the induction of T-cell mediated acute murine GI-GvHD leads to an enhanced intestinal permeability characterized by high expression of TNF-α leading to a downregulation and localization shift of the tight junction protein occludin, whereas ZO-1 protein expression and localisation within the tight junction remains unchanged. These observations are associated with morphologic changes of the tight-junctions. Further investigations are needed for a better understanding of the pathophysiological regulation of tight-junctions within the intestinal T-cell mediated acute GI-GvHD following bone marrow transplantation to find opportunities to influence the barrier defect and therefore these gastrointestinal complications.

## Competing interests

The authors declare that they have no competing interests.

## Authors' contributions

RN, JLG, ES, and MLK and ME carried out the experimental studies and drafted the manuscript. RN, RH, JH, BB, PR and SS participated in the design of the study and performed the statistical analysis. PR and AA conceived of the study, and participated in its design and coordination and helped to draft the manuscript. All authors read and approved the final manuscript.

## Pre-publication history

The pre-publication history for this paper can be accessed here:

http://www.biomedcentral.com/1471-230X/11/109/prepub
